# Our Young Dental Patients

**Published:** 1880-01

**Authors:** Charles E. Francis

**Affiliations:** New York


ARTICLE VI.

### OUR YOUNG DENTAL PATIENTS.

BY CHARLES E. FRANCIS, D. D. S., NEW YORK.

It is a noteworthy fact that a goodly proportion of our patients are children, and the ratio seems to be ever on the increase. A quarter century ago comparatively few children under ten years of age had the benefit of the dentist's care, except, perhaps, for the purpose of having loose or aching teeth removed. The deciduous teeth were considered of trifling value, and, as their mission was of so temporary a character, it mattered but little whether they remained in the mouth until forced out by the natural eruption of their more substantial successors, or were removed by aid of forceps long before the new-comers were due. Many individuals imagined that the "milk teeth" were without roots—mere crowns or "shells" attached only to the gums. Of course, these teeth received little attention, and scarcely any regard was paid to their preservation. The idea of filling them when attacked by caries was considered absurd, if, indeed, such an idea was considered at all. When sufficiently decayed to be very painful, the only resort was to have them extracted.

Some twelve years ago, a lady patient, whose teeth had suffered for want of attention during her youth, and being desirous that her little ones should be better cared for and escape evils that she had experienced, asked what could be done for her little son, scarcely four years of age, whose deciduous molars were already beginning to decay. Upon being advised to have them filled, she, upon her return home, consulted her husband, a man of excellent physical development, and who never had occasion to visit a dentist, requesting his consent to have the child's teeth attended to. He, however, at once objected, declaring it a "ridiculous and extravagant notion" on her part, and would not permit it.

One cold winter morning, a few months later, this husband and father came to my office, at an early hour, bringing with him his boy. The little fellow had for several days and nights been suffering with toothache, getting little or no sleep himself, and, by his moaning and crying, preventing also his parents from sleeping. Both sides of his face were much swollen, and tears were streaming down the little plump cheeks. Prompted by a natural impulse, I at once administered a sharp rebuke to the parent for so neglecting the little one, at the same time telling him plainly the evil results of such negligence. He admitted his error, as he viewed with pitying eye the little sufferer.

How few are aware of the dire consequences of such sins of omission! Needless tortures of toothache and loss of sleep, both taxing seriously the nervous organism; loss of natural means of mastication, forcing upon the stomach food imperfectly comminuted, thus impairing the integrity of that receptacle, and making digestion difficult and incomplete; allowing the breath to become offensive, and the oral fluids to become vitiated ; resorting to the premature removal of the temporary teeth, with the risk of contracted dental arches, and second dentition irregular and diseased. A little timely care and attention will oftentimes save serious suffering, trouble, expense and *teeth.*

When, however, children are taken to the dentist they should be kindly treated. First impressions are apt to be lasting, and if children receive gentle treatment at first, and their confidence fully gained, there will be comparatively little trouble with all future operations. If, on the other hand, children become intimidated or suffer severe pain on their first visit to the dentist, they will seldom forget it, and will always fear to go again.

A lady recently said to me that when she was ten years of age, her father, being anxious to have her teeth well cared for, took her to the office of his dentist, requesting that his child should have whatever attention was required, and then left her in the dentist's charge. The latter, on examining the child's mouth, decided that all the sixth year molars must be extracted, and, despite her protestations and pleadings, a muscular female servant was summoned to hold the child, while the four huge molars, one after the other, were dragged out. The operation so frightened her that for years after she avoided dentists, and her teeth became nearly all ruined for want of attention.

To operate for children of course requires *patience*, but the more patient we are with the *child*, the less will our patience be taxed as we advance in years.

